# Visible-Light Photocatalytic Reduction of Aryl Halides as a Source of Aryl Radicals

**DOI:** 10.3390/molecules27175364

**Published:** 2022-08-23

**Authors:** Jihong Lan, Rongxiang Chen, Fangfang Duo, Menghui Hu, Xiaoyan Lu

**Affiliations:** 1 School of Chemistry and Materials Engineering, Xinxiang University, Xinxiang 453003, China; 2 School of Pharmacy, Xinxiang University, Xinxiang 453003, China; 3 College of Chemistry and Chemical Engineering, Luoyang Normal University, Luoyang 471934, China

**Keywords:** visible light, aryl halides, photoinduced electron transfer, reduction, aryl radicals

## Abstract

Aryl- and heteroaryl units are present in a wide variety of natural products, pharmaceuticals, and functional materials. The method for reduction of aryl halides with ubiquitous distribution is highly sought after for late-stage construction of various aromatic compounds. The visible-light-driven reduction of aryl halides to aryl radicals by electron transfer provides an efficient, simple, and environmentally friendly method for the construction of aromatic compounds. This review summarizes the recent progress in the generation of aryl radicals by visible-light-driven reduction of aryl halides with metal complexes, organic compounds, semiconductors as catalysts, and alkali-assisted reaction system. The ability and mechanism of reduction of aromatic halides in various visible light induced systems are summarized, intending to illustrate a comprehensive introduction of this research topic to the readers.

## 1. Introduction

Aryl groups are widely used intermediates in organic synthesis, especially in inter functional group transformations and C-C bonding reactions [1]. Introduction of (hybrid)aryl groups into molecular scaffolds is one of the basic chemical transformation processes in the synthesis of natural products, drugs, or functional materials [2,3,4]. Over the past few decades, arylation reactions have been mainly performed by transition metal catalysis, but this process suffers from several problems, such as the need for expensive transition metal catalysts, air sensitive ligands, and stoichiometric metal reductants and other harsh reaction conditions [5]. The visible light is an abundant, clean, and renewable energy. Visible-light-driven arylation provides a cheap, efficient, and environmentally safe method, which is the research focus in recent years. An enormous amount of literature for visible-light-driven photocatalytic reduction of aromatic compounds to prepare aryl radicals under mild conditions has been reported [6,7,8,9,10,11]. For example, König summarized visible-light-driven arylation reactions of aryl diazonium salts, aryl iodonium salts, aryl halides, and other substrates [7]. Huang reviewed the reactions of aryl diazonium salts as substrates to construct C-C, C-B, C-N, C-S and C-P bonds under visible light conditions [10]. Kvasovs and Gevorgyan reviewed the photocatalytic systems for the photocatalytic generation of aryl radicals using aryl diazonium salts, aryl halides, aryl triflates, and aryl carboxylic acids as precursors and presented their modern application examples [11].

**Figure 1:**
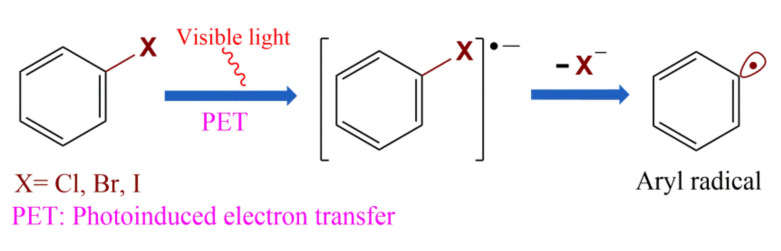
Generation and transformations of aryl radicals.

Among the numerous precursors of aryl radicals [12,13,14,15,16,17], aryl halides are the most readily available and least expensive arylation reagents [8]. However, no systematic review has been reported on the preparation of aryl radicals by visible light-induced reduction of aryl halides. Due to the extreme redox potentials of aryl halides, their range of applications is limited by the reduction ability of excited photosensitizers and the visible light energy [18,19,20]. The general process of visible-light-driven reduction of aryl halides to aryl radicals initiates by visible light-induced electron transfer from excited photocatalyst to aryl halide to generate an aryl halide radical anion, closely followed by the halide negative ion-mediated cleavage of carbon–halide bond, leaving halide ions (X^–^) to generate a highly reactive aryl radical intermediate that can be used to construct various classes of aryl compounds (Figure 1). Since aryl fluoride free radical anion intermediates, which are generated through photoinduced electron transfer from excited photocatalyst to the aryl fluoride compounds, can be attacked by electrophiles to leave fluoride ions and generate various aryl compounds [21], there is no formation of aryl radical intermediates in the whole process, so visible light-induced photocatalytic reduction of aryl fluorides is not discussed in this review.

According to the mechanism of visible-light-driven reduction of aryl halides, the highly efficient visible light photosensitizer is the key factor of aryl halides reduction. At the same time, the study of reaction mechanism is helpful to understand the visible-light-driven reduction process of aromatic halogen, including the formation of transition states, bond breaking and formation, etc. According to these, the high-efficiency photocatalysts can be designed, and the reaction conditions can be adjusted to improve the yield of products. Based on this, this review systematically summarizes the reaction systems of the reduction of aromatic halides by visible light-induced electron transfer with metal complexes, organic compounds, semiconductor as catalyst, and alkali-assisted reaction system focusing on the ability and reaction mechanism of the reduction of aromatic halides in various systems. Finally, the summary and prospect of this research topic are given.

## 2. Visible-Light-Driven Reduction of Aryl Halides with Metal Complexes as Photocatalysts

### 2.1. Conventional Photoinduced Electron Transfer

**Figure 2:**
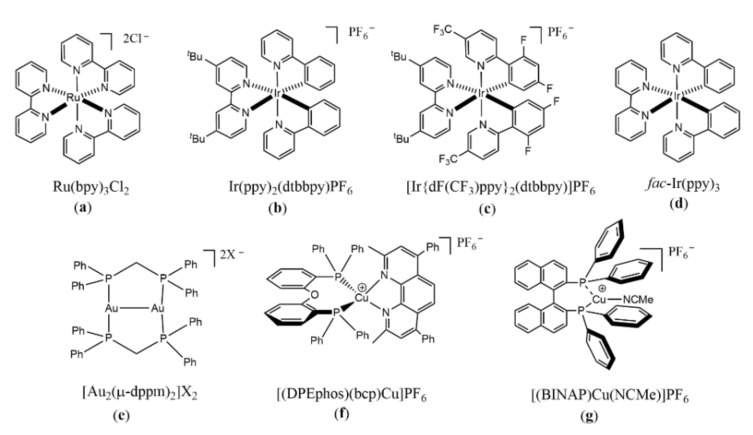
The structure of organometallic photocatalysts. (**a**) Ru(bpy)_3_Cl_2_; (**b**) Ir(dtbbpy)(ppy)_2_PF_6_ (**c**) [Ir{dF (CF_3_)ppy}_2_(dtbbpy)]PF_6_; (**d**) *fac*-Ir(ppy)_3_; (**e**) [Au_2_(μ-dppm)_2_]X_2_; (**f**) [(DPEphos)(bcp)Cu]PF_6_; (**g**) [(BINAP)Cu(NCMe)]PF_6_. (bpy = 2,2′-bipyridyl, dtbbpy = di-tert-butyl-2,2′-bipyridine, ppy = 2-phenylpyridinated; ppm = bis(diphenylphosphanyl)methane; DPEPhos: bis[2-(diphenylphosphino)phenyl] ether; bcp: 2,9-dimethyl-4,7-diphenyl-1,10-phenanthroline; BINAP: 2,2-bis (diphenylphospino)-1, 1′–binaphthyl; NCME: methyl cyanide).

**Figure 3:**
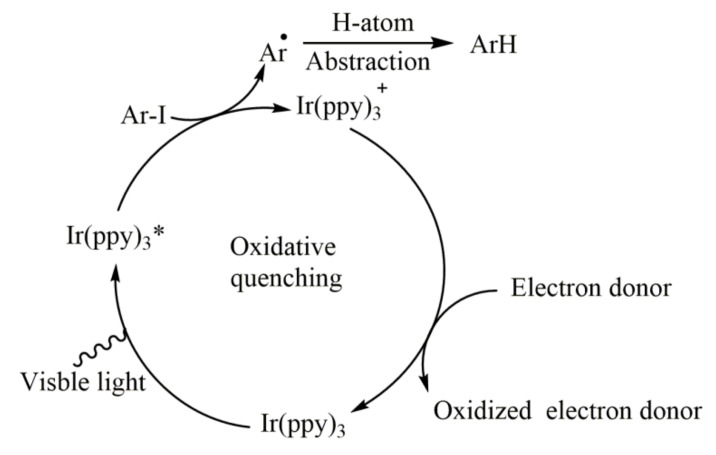
The mechanism of visible-light-driven aryl iodide reduction with *fac*-Ir(ppy)_3_ as photosensitizer. Reprinted with permission from Ref. [18]. Copyright 2012, Nature Publishing Group.

At present, the most widely used organometallic complexes are Ir complexes and Ru complexes, and their reduction potentials depend on central metals and ligands. Ru(bpy)_3_Cl_2_ (Figure 2a) [22], Ir(dtbbpy)(ppy)_2_PF_6_ (Figure 2b) [23,24,25], [Ir{dF (CF_3_)ppy}_2_(dtbbpy)]PF_6_ (Figure 2c) [24], and *fac*-Ir(ppy)_3_ (Figure 2d) [18] are commonly used photosensitizers for the reduction of aromatic halides. Among them, *fac*-Ir(ppy)_3_ has the highest reduction potential and can effectively reduce and cleave the carbon halogen bonds of inactive alkyl, alkenyl, and aryl iodides to generate corresponding radicals (Figure 3), which can generate C-H products, or undergo intramolecular cyclizations to provide a variety of cyclic scaffolds [18]. In general, ruthenium and iridium composite catalysts can only reduce the highly active weak aromatic halogen bonds, such as electron-deficient aryl iodides. At the same time, dimeric gold complexes (Figure 2e) can efficiently generate aryl radical intermediates by reducing inactivated aryl bromine under mild conditions [26]. Meanwhile, Wang found that the heterogeneous Ag(0) in monatomic dispersion state can realize the deiodination-arylation, the selective hydrogenation, and boration of aryl iodides through single electron transfer process under visible light conditions [27,28].

**Figure 4:**
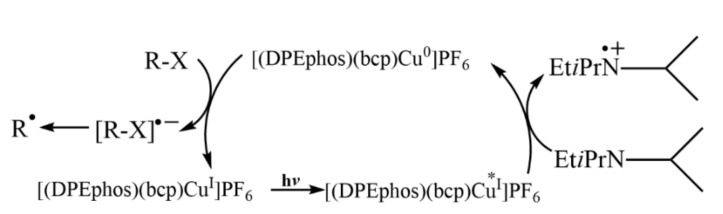
The mechanism of visible-light-driven aryl halides reduction with [(DPEphos)(bcp)Cu]PF_6_ as photosensitizer. Reprinted with permission from Ref. [29]. Copyright 2017 American Chemical Society.

**Figure 5:**
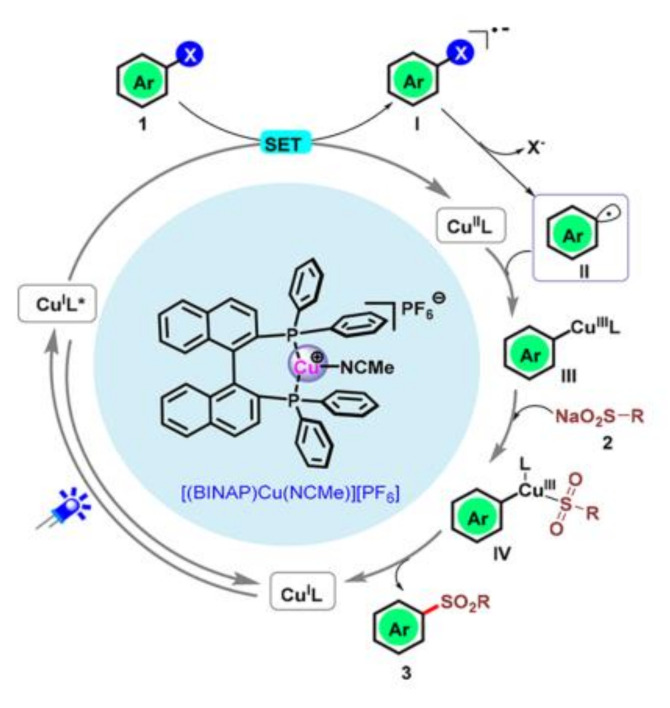
The mechanism of visible-light-driven aryl halides reduction with [(BINAP)Cu(NCMe)]PF_6_] as photosensitizer. Reprinted with permission from Ref. [30]. Copyright 2021, American Chemical Society.

**Figure 6:**
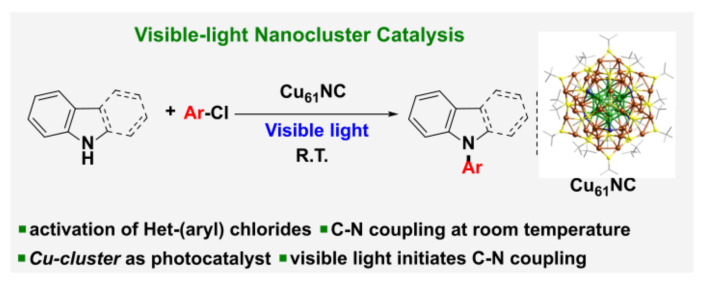
Visible-light copper nanocluster [Cu_61_(S*^t^*Bu)_26_S_6_Cl_6_H_14_](Cu_61_NC) catalysis for the C−N coupling of aryl chlorides. Reprinted with permission from Ref. [31]. Copyright 2022, American Chemical Society.

In addition to those precious metal complexes, copper complexes can also be used to reduce aryl halides. Under visible light irradiation, a series of inactivated aryl iodides and aryl bromides could be reduced to aryl radicals by [(DPEphos)(BCP)Cu]PF_6_ (Figure 2f) with the assistance of amine through a Cu(I)/Cu(I)*/Cu(0) catalytic cycle and then undergo hydrogenation or coupling reactions [29] (Figure 4). Under visible light irradiation, Cu(I) complex [(BINAP)Cu(NCME)]PF_6_] (Figure 2g) generated in situ, was excited to form CuL*, which could reduce aryl iodide and aryl bromide through electron transfer process to aryl radical, and CuL* was oxidated to LCu(II). Then, aryl radical reacted with sodium alkyl sulfonate through oxidative addition and reductive elimination in the presence of LCu(II) to produce phenyl sulfone and LCu(I), completing the catalytic cycle (Figure 5) [30]. Latterly Bakr and Rueping discovered that a single electron transfer (SET) process between the visible-light-excited copper nanocluster-based complex [Cu_61_(S*^t^*Bu)_26_S_6_Cl_6_H_14_](Cu_61_NC) and aryl halides (including aryl chlorides) enabled the C−N-arylation reaction in good yields (Figure 6) [31].

### 2.2. Sensitization-Initiated Electron Transfer

**Figure 7:**
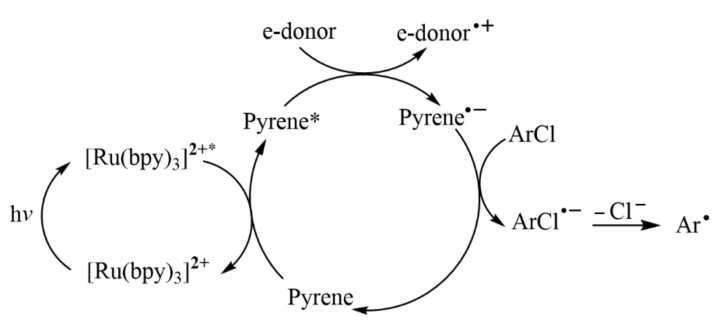
Proposed mechanism for two-center photo-redox catalysis with Ru(bpy)_3_Cl_2_/pyrene. Reprinted with permission from Ref. [35]. Copyright 2017, WILEY-VCH Verlag GmbH & Co. KGaA, Weinheim.

The reduction ability of most of these metal complexes is limited, which can only reduce aromatic iodine and aromatic bromide. Aromatic halides can be activated and reduced by sensitization-initiated electron transfer (SenT-ET) for photocatalysis [32,33]. In this process, by collecting light energy through highly light-absorbing metal complexes, and then transferring the excitation energy to a redox catalyst that does not absorb visible light via an energy transfer process, the reduction ability can be significantly enhanced [34]. The visible-light-driven reduction of challenging aryl chloride compounds can be achieved by the double center photocatalytic redox system with metal complexes. For example, when Ru(bpy)_3_Cl_2_ absorbed visible light and transferred energy to pyrene, which was a polycyclic aromatic hydrocarbon (PAH) capable of redox reactions, activated aryl chloride compounds could be reduced (Figure 7) [35]. Subsequently, Ceroni and Balzani further improved this mechanism [36]. In 2020, Moore and coworkers disclosed the mechanism of SenI-ET reaction for the C−H arylation of activated aryl halides using [Ru(bpy)_3_]^2+^ and PAH (such as pyrene), by characterizing the kinetic behavior of the different excited-state species involved [37]. However, the key catalytic species pyrenyl radical anion (Py^•–^) was not all detected [35,36,37].

**Figure 8:**
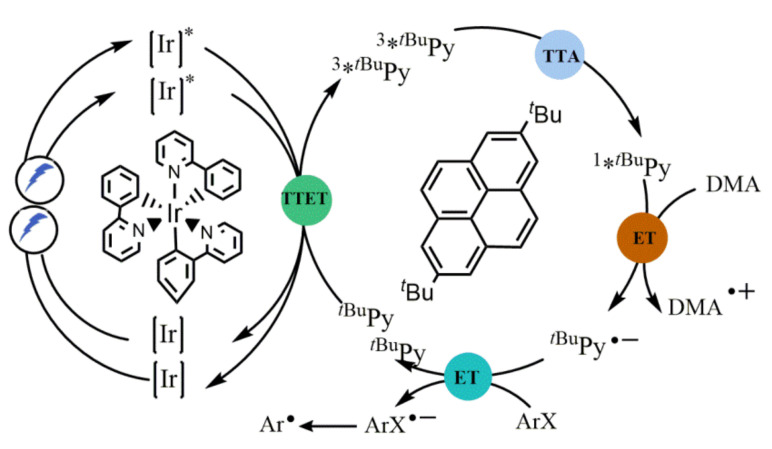
Mechanism for sensitization-initiated electron transfer (SenI-ET) with the *fac*-[Ir(ppy)_3_]/*^t^*^Bu^Py/DMA combination. Reprinted with permission from Ref. [38]. Copyright 2021, Royal Society of Chemistry.

Wenger reported a new two-center photoredox reaction system of *fac*-[Ir(ppy)_3_]/*^t^*^Bu^Py/DMA (ppy = 2-phenylpyridine; *^t^*^Bu^Py = 2,7-di-*tert*-butyrypyrene; DMA = *N*,*N*-dimethylformamide), which was not only suitable for photocatalytic aryl dehalogenations but also for pinacol couplings and detosylation reactions (Figure 8) [38]. Notably, they detected all relevant reaction intermediates directly by transient absorption and emission spectroscopy and determined the mechanism of sensitization-initiated electron transfer works for this system. Combining with spectral data, they determined that the reaction was consisted of triplet–triplet energy transfer (TTET), triplet–triplet annihilation upconversion (TTA-UC), pyrenyl radical anion formation, and substrate activation (Figure 8). The sensitization-initiated photoredox process makes it possible to activate stable chemical bonds using extremely reductive molecules that do not absorb visible light.

## 3. Visible-Light-Driven Reduction of Aryl Halides with Organic Complexes as Photocatalysts

### 3.1. Single Photon Excitation

**Figure 9:**
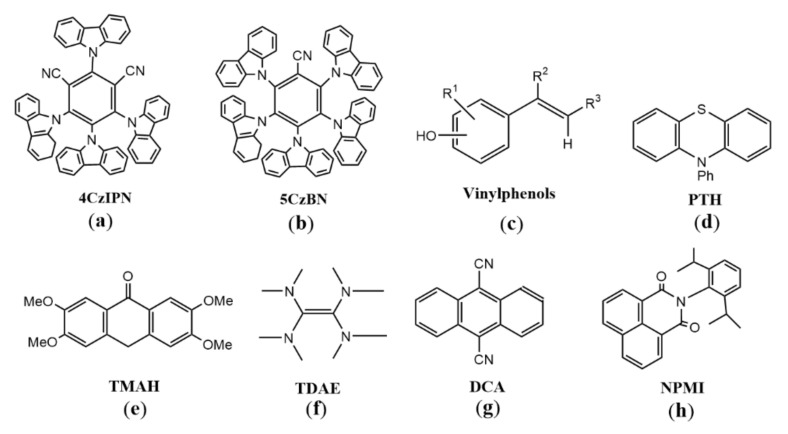
Organic photocatalysts involving single-photon excitation. (**a**) 4CzIPN: 1, 2, 3, 5-tetrakis (carbazol-9-yl)-4, 6-dicyanobenzene; (**b**) 5CzBN: carbazolyl cyanophenyl; (**c**) Vinylphenols; (**d**) PTH: *N*-phenylphenothiazine; (**e**) TMAH: 2, 3, 6, 7-tetramethoxyanthrone; (**f**) TDAE: tetramine (dimethylamine) ethylene; (**g**) DCA: 9, 10-dicyano-anthracene; (**h**) NPMI: 1, 8-naphthimide.

**Figure 10:**
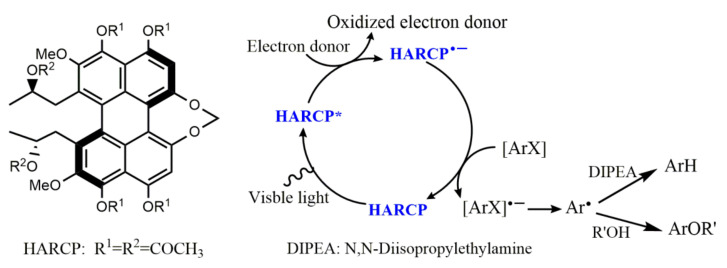
The structural formula of HARCP and HARCP photoactivation of aryl halides with PET. Reprinted with permission from Ref. [42]. Copyright 2021, Elsevier Inc.

Compared with metal complex photosensitizers, organic photosensitizers have the advantages of environmental friendliness, low price, and sustainability. However, organic photosensitizers have weak spin orbit coupling and short excited state lifetime. In 2012, the Adachi team synthesized high-efficiency organic light-emitting diodes using delayed fluorescence by combining spatially separated donors and receptors. The donor and acceptor parts were placed orthogonally through the steric hindrance, and the highest occupied molecular orbital and the lowest unoccupied molecular orbital were located on the donor and acceptor parts, respectively, so that the energy difference between singlet and triplet states was very small. Compared with organometallic photocatalysts, the organic molecule 4CzIPN (Figure 9a) could easily cross electrons from singlet to triplet through a small energy gap, and the triplet life was longer [39]. Inspired by this, many researchers synthesized organic molecules with similar structures and applied them as catalysts in photoredox chemistry [40,41]. With the assistance of electron donors, organic photosensitizers can realize the visible-light-driven reduction of activated halogenated aromatic compounds. For example, according to the principle of structure–property relationship, Huang developed a new type of organic perylene photoreductant, hexacetyl-reduced cercosporin (HARCP). HARCP* was produced when HARCP was excited by visible light, and then reduced aryl bromine and activated aryl chloride compounds through photoinduced electron donors to construct aryl hydrides and aryl phenols under mild conditions [42] (Figure 10). In addition, carbazolyl cyanophenyl (5CzBN) (Figure 9b) [43] could reduce activated aryl chloride to aryl hydride through photoinduced electron donor under visible light irradiation. Recently, Wang and Chen reported that under blue light irradiation, *N*-heterocyclic nitrenium (NHN) salt, as a catalytic electron acceptor for the photoactivation of charge transfer complexes, could catalyze the generation of aryl free radicals from aryl chlorine through single electron transfer in present of electron donor [44].

**Figure 11:**
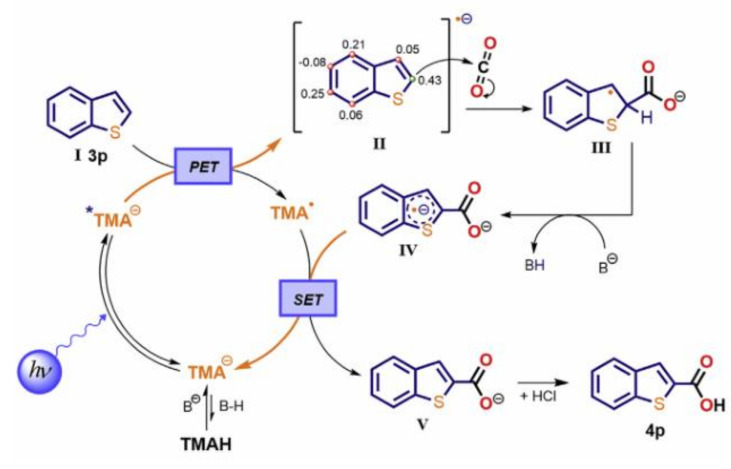
Proposed mechanism for the C-H carboxylation of (hetero)arenes with TMAH as the photosensitizer. Reprinted with permission from Ref. [48]. Copyright 2020, Elsevier Inc.

There are also some visible-light-driven photosensitizers whose excited states are strongly reductive and can directly reduce inactivated aromatic halides to generate aromatic radicals without sacrificing any reductant. Vinylphenolic anions in the excited state (Figure 9c) could reduce a wide range of aryl- and heteroaryl halides (iodides, bromides, and chlorides) without sacrificing any reductant, and then the resulting aryl radical coupled with another vinylphenol molecule to provide Heck-type aryl products in a region-specific and stereoselective manner [45]. Jui used *N*-phenylphenothiazine (PTH) (Figure 9d) as the visible-light-driven photocatalyst to reduce aryl halides, including aryl chloride without the assistance of electron donors, and then the generated aryl radical underwent nucleophilic addition with ethylene amine derivatives to fully region-controlled aryl ethylamines [46]. König used 9-anthranone anion as visible-light-driven photocatalyst that did not require electron donor assistance to reduce various aryl (heteroaryl) chlorides under blue light excitation, providing corresponding aryl products at moderate to extremely good yields [47]. With the assistance of bases, 2,3,6,7-tetramethoxyanthrone anion (TMA^−^) (Figure 9e) could reduce aromatic hydrocarbons without electron donors to the corresponding aromatic radical anion, which then reacted with CO_2_ to obtain the corresponding carboxylic acid, providing a new opportunity for the valorization of aromatic hydrocarbons (Figure 11) [48]. Guan and Shang found that *o-*phosphinophenol and *o-*phosphinothiophenol were photocatalysts with strong reduction ability, which could activate aryl chloride and bromide under visible light for boration, arylation and phosphorylation [44]. They also found that *o-*diphenylphosphine substituents could lead to narrow optical gaps and promote the cross entry into the triplet state between systems, thus promoting the role of phenolate and thiophenol as effective visible light photo redox catalysts. Therefore, the synthesis of modified phenols and thiophenols as photocatalysts will have broad prospects.

**Figure 12:**
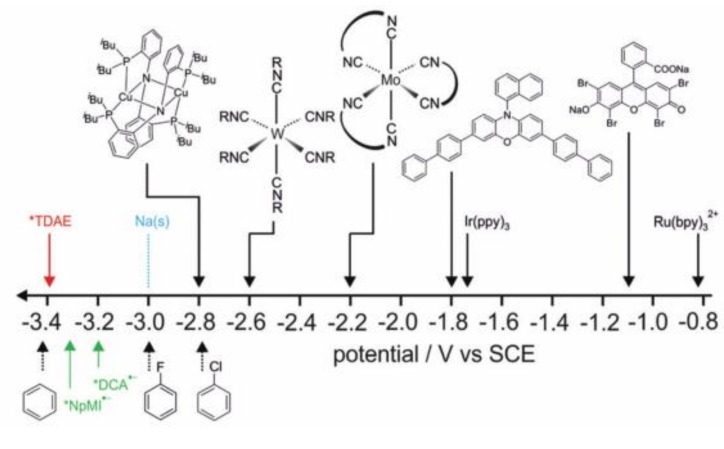
Overview of some of the most potent excited-state photoreductants operating on the basis of single excitation with visible photons known to date. Reprinted with permission from Ref. [57]. Copyright 2020, the Royal Society of Chemistry and Owner Societies.

**Figure 13:**
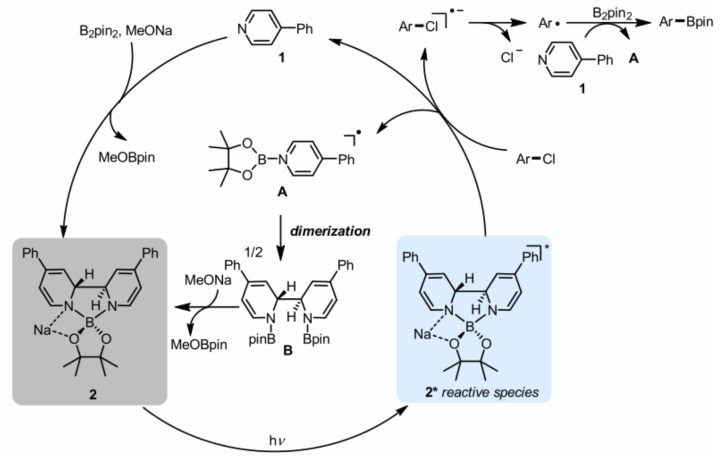
Borylation mechanism of aryl chloride. Reprinted with permission from Ref. [58]. Copyright 2019, American Chemical Society.

In addition, the organic electron donor is a strong single-electron transfer reagent in the organic synthesis [49]. It is found that the reduction ability of electron donors can be significantly enhanced by photoexcitation [50]. Thus, extremely negative excited state reduction potentials can be obtained by photoexcitation of suitable electron donors for reductive dehalogenation of aryl halides. For example: The commercially available electron donor tetramine (dimethylamine) ethylene (TDAE) (Figure 9f) had an excited oxidation potential of −3.4 V when it was excited by LEDs at 440 or 390 nm, which was very favorable compared to the strongest known photoreductants of visible light single excitation (Figure 12) [51,52,53,54,55,56], and it could reduce stoichiometric aryl chlorides and fluoride [57]. However, TDAE^•+^ could not be oxidized by normal sacrificial electron donors to regenerate TDAE, so TDAE cannot be used in catalytic cycles. An organic super electron donor of divalent diboron/methoxide/4-phenylpyridine with an excitation potential of −3.5 V was in situ generated under blue light, and its excited state lifetime was about three times that of TDAE. This organic super electron donor could reduce the inactivated aromatic chloride to generate aromatic radicals, which reacted with diboron to produce aryl boronate (Figure 13) [58]. Currently, the excited state energies of most other (non-commercial) organic super electron donors are unknown and their excited state potentials cannot be estimated. Therefore, further exploration of these substances in terms of photophysics, photochemistry, and excited state properties is expected to complete more challenging reactions.

**Figure 14:**
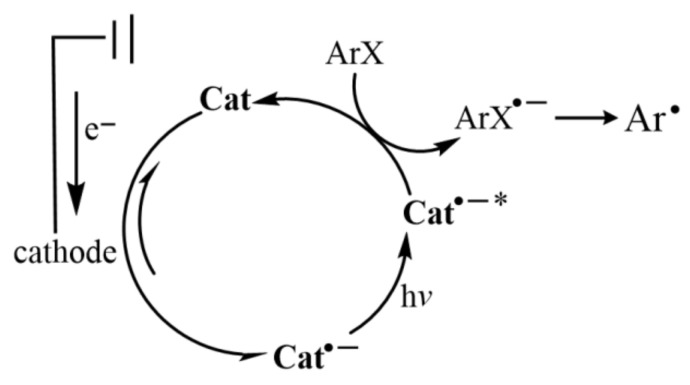
Proposed catalytic cycle of electro-photo-catalytically generated aryl radicals. Reprinted with permission from Ref. [56]. Copyright 2020, American Chemical Society.

In addition, photochemical active radical anions can be generated by electrochemical cathodes, and then highly reductive excited photoreductants can be obtained by visible light excitation to reduce aryl halides to active aryl radicals (Figure 14). Lin and Lambert’s research group obtained the excited 9, 10-dicyano-anthracene (DCA) (Figure 9g) radical anion with reduction potential up to −3.2V through the coordinated cathodic reduction and visible light excitation, which could reduce various aryl chlorides [55]. Wickens reported that 1,8-naphthimide (NPMI) radical anion generated by electrochemical cathodic reduction (Figure 9h) could reduce different substituted aryl chlorides after blue light excitation to construct carbon–carbon and carbon–heteroatomic bonds [56]. The development of this concept will promote the development of organic reactions requiring extreme redox capacity, such as the reduction of aromatic halides.

### 3.2. Two-Photon Excitation

**Figure 15:**
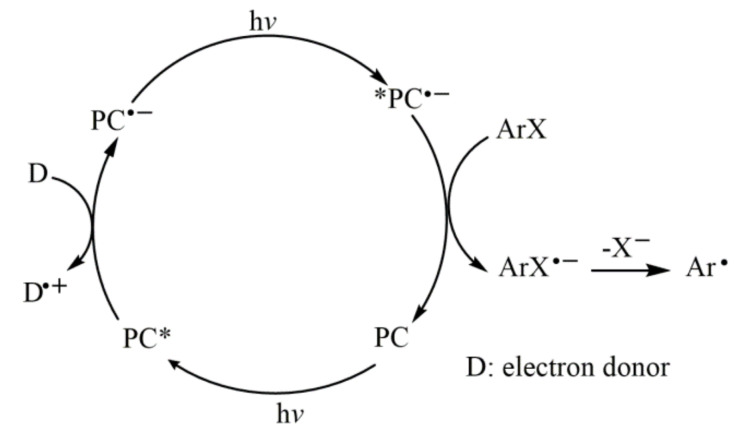
Proposed mechanism of reduction of aryl radicals by consecutive visible-light-induced electron transfer processes.

The reductive cleavage of inactive aryl bromide/chloride by photocatalytic single electron transfer is beyond the range of visible spectrum [41], which greatly limits the functionalization of aryl groups. In addition to UV treatment [59,60,61] and electrochemical reduction-single electron transfer [55,56], two-photon excitation can alleviate the above problems. Two-photon excitation is that the visible light photosensitizer forms a new activation mode through twice light excitation and twice photoinduced electron transfer (PET), which greatly improves the reducibility of the photocatalyst (Figure 15) and expands the application range of aromatic halides.

**Figure 16:**
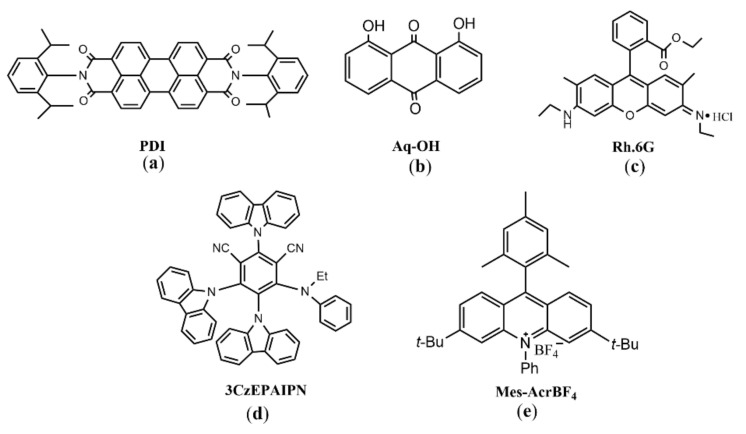
Organic photocatalysts involving two-photon excitation (**a**) PDI: Perylene bisimide; (**b**) Aq-OH: 1,8-dihydroxyanthraquinone; (**c**) Rh.6G: Rhodamine 6G; (**d**) 3CzEPAIPN: 2,4,5-tri (9H-carbazol-9-yl)-6-(ethyl (phenyl) amino) *m*-benzonitrile; (**e**) Mes-AcrBF_4_: 3-(Tert-butyl)-6-isopropyl-9-mesityl-10-phenylacridin-10-ium tetrafluoroborate).

**Figure 17:**
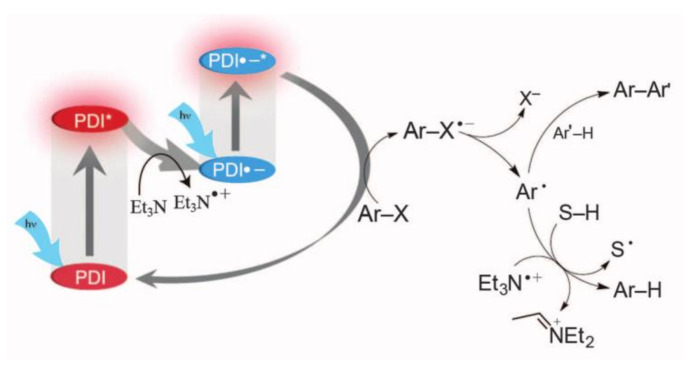
Organic proposed mechanism of reduction of aryl halides with PDI by consecutive visible light-induced electron transfer processes. Reprinted with permission from Ref. [19]. Copyright 2014, The American Association for the Advancement of Science.

Perylene bisimide (PDI) (Figure 16a), which accumulated energy through two solar excitation, could reduce stable aryl chloride to highly reactive aryl radicals. These aryl radicals were captured by hydrogen atom donors or used to form carbon–carbon bonds [19] (Figure 17). However, the reaction system has problems, such as weak activation ability and poor solubility of PDI. Duan solved these shortcomings by combining PDI into metal organic polymer (Zn-PDI). After two visible light excitations, it could efficiently catalyze the reduction of aryl chloride [62]. Since both organic molecules and metal organic polymers allow for further fine-tuning and optimization, the reaction system provides a reference for the design of high activity heterogeneous photocatalysts. Wangelin and Pérez-Ruiz found a strongly reduced excited anion DCA^–^* (Figure 9g), which was generated through two photon activation processes, could cleave the inactive aryl chloride bond. The generated aryl radicals could further form C-H, C-C, C-P, C-S and C-B bonds to construct new aryl compounds [63]. The 1,8-dihydroxyanthraquinone (Figure 16b) radical anion AQ-OH^−^*, which was generated by primary photoinduced electron reduction and secondary visible light excitation of AQ-OH, could reduce aryl halides, including aromatic chlorine. The generated aryl radical could further form C-H or C-C bonds compounds [64]. König used Rhodamine 6G (Rh.6G) (Figure 16c) as a photosensitizer, and Rh.6G^–^*,which was generated after two times of blue light excitation, could reduce aromatic bromine to the aryl radical; they then prepared arylphosphonate by Arbuzov reaction with trialkyl phosphites [65].

**Figure 18:**
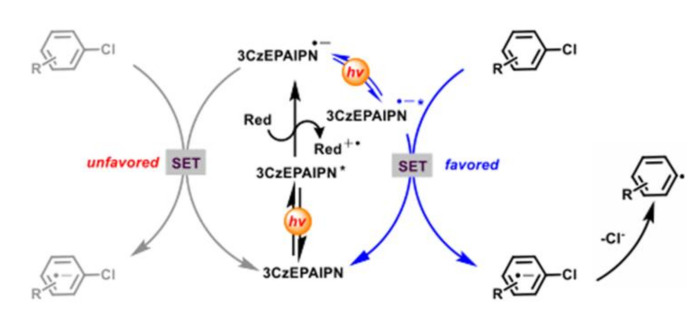
Proposed mechanism of reduction of aryl radicals by consecutive visible light-induced electron transfer processes with 3CzEPAIPN. Reprinted with permission from Ref. [66]. Copyright 2021, American Chemical Society.

These above two-photon excitation process could only reduce electron-deficient aryl chloride efficiently [19,63,64,65]. In recent years, photosensitizers with more negative reduction potentials, which are excited by two photons, can efficiently reduce electron-rich aryl chlorides. For example, after two visible light excitations, the excited 2,4,5-tri (9H-carbazol-9-yl)-6-(ethyl (phenyl) amino) *m*-benzonitrile (3CzEPAIPN^–^*) (Figure 16d) was generated with an excited state lifetime of 12.95 ns, which could efficiently reduce various aryl chlorides (*E*_red_ ≈−1.9 to −2.9 V vs SCE) to prepare aromatic C-B, C-P and C-C bond compounds (Figure 18) [66]. Acridinium salts Mes-Acr^+^BF_4_^−^ (Figure 16e) underwent a light excitation to form an acridine radical Mes-acr^•^. After another photoexcitation, Mes-acr^•^* was generated with a reduction potential of −3.36V vs. SCE, which can effectively reduce various aromatic chlorines [67]. However, although two-photon excited redox process can make full use of solar energy and have a strong redox ability, more side reactions will occur, compared with single photon excitation [68]. 

## 4. Others

### 4.1. Visible Light-Induced, Base-Promoted Reduction of Aryl Halides

**Figure 19:**
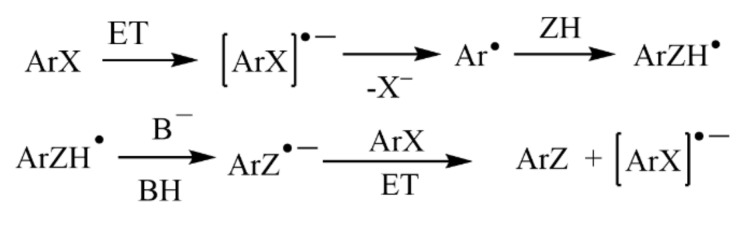
The mechanism of base-promoted homolytic aromatic substitutions. Reprinted with permission from Ref. [69]. Copyright 2013, American Chemical Society.

**Figure 20:**
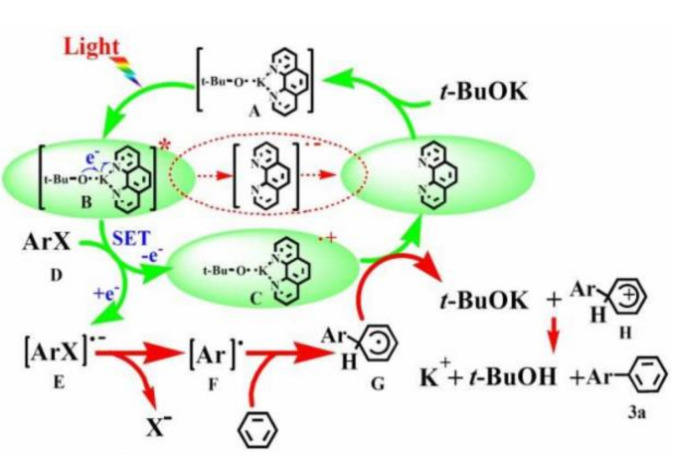
Proposed plausible mechanism of visible light photoredox catalyzed aryl halides reduction using base and nitrogen heterocycles as a promoter. Reprinted with permission from Ref. [70]. Copyright 2015, American Chemical Society.

**Figure 21:**
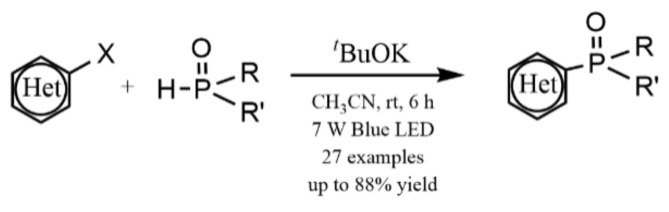
Visible light-promoted phosphinylation of heteroaryl halides in the presence of *t*-BuOK. Reprinted with permission from Ref. [71]. Copyright 2018, American Chemical Society.

**Figure 22:**
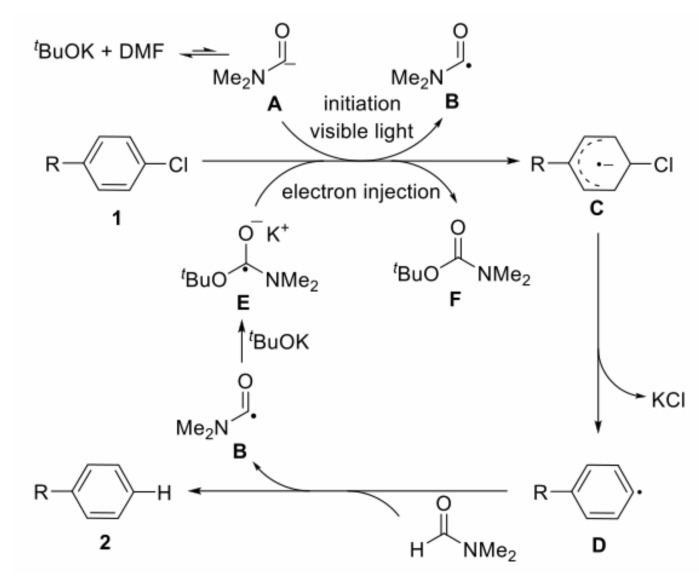
Proposed mechanism of visible-light-induced, base-promoted dehalogenation of aryl halides. Reprinted with permission from Ref. [78]. Copyright 2020, American Chemical Society.

Visible light-induced, base-promoted free radical reaction can take place at room temperature without transition metals and additional ligands. This type of reaction has gradually attracted the attention of chemists because of high efficiency, few side reactions, and controllability. Visible light-induced, base-promoted electron transfer can also be used for aryl halide reduction. For example, Rossi reported homolysis of aryl halides (chlorine, bromine, iodine) to realize C-H arylation reaction at room temperature under visible light-induced, potassium *tert*-butanol-promoted conditions [69] and proposed the mechanism of aryl homolytic reaction promoted by a base (Figure 19). [ArX]^•–^ was formed firstly by photoinduced electron transfer, and then [ArX]^•−^ broke to form [Ar]^•^ and [X]^–^, [Ar]^•^ coupled with ZH to produce [ArZH]^•^, [ArZH]^•^ reacted with alkali to [ArZ]^•–^. At last, the electron was transferred from [ArZ]^•–^ to ArX, forming the products ArZ and [ArX]^•–^, which proceeded to the next cycle. In 2015, Yuan reported a new mode for the reduction of aromatic halides (chlorine, bromine, iodine) to prepare diaryl compounds by using a photosensitive complex of potassium *tert*-butoxide (*t*-BuOK) and nitrogenous heterocyclic ligands via visible light excitation (Figure 20) [70]. In this reaction system, *t*-BuOK and 1,10-phenanthrene formed a five-membered ring compound. Under the excitation of visible light, electrons in the compound were transferred from the electron donor *t*-BuOK to the electron-deficient 1,10-phenanthrene ligand via potassium atoms, and 1,10-phenanthrene received electrons to form stable radical anions. The 1,10-phenanthrene radical anions could reduce aromatic halides by single electron transfer to aryl radicals, which could be coupled with benzene to generate diphenyl compounds. In 2018, Yu reported that the visible-light-driven phosphinylation of heteroaryl halides (chlorine, bromine, iodine) could be realized in the presence of *t*-BuOK [71] (Figure 21). UV-vis studies showed that neither the substrate nor *t*-BuOK could absorb visible light. It was possible that a charge transfer complex was formed between the substrate and *t*-BuOK, with K^+^ acting as a bridge between pyridine and *tert*-butanol anions, which was similar to studies on *t*-BuOK as an electron donor [70,72,73,74,75,76,77]. In 2020, Kang proposed visible-light-induced, *t*-BuOK-promoted dehalogenation and hydrogenation of aryl chloride in *N*, *N*-dimethylformamide, without additional hydrogen sources [78]. The reaction mechanism was proposed on the basis of a free radical capture experiment and deuteration experiment. Under the promotion of visible light, electrons were transferred from deprotonated DMF (A) to aryl halide, forming radical B and radical anion C. Halogen ion was removed from C forming aryl radical D which took a hydrogen atom from DMF to generate dehalogenated product and B. B was attacked by *t*-BuOK to generate anion E, which was the main electron donor for the further reaction (Figure 22).

### 4.2. Visible-Light-Driven Reduction of Aryl Halides with Semiconductors as Photocatalysts

Most of the above reactions are completed under homogeneous conditions; the problem of catalyst recovery and reuse urgently needs to be solved. Catalytic reduction of aryl halides by heterogeneous semiconductors can avoid the above problems. The mechanism of the photocatalytic reaction by semiconductors is as follows: under the excitation of the visible light, electrons absorbing photons in the valence band (VB) are excited to jump from the VB to the conduction band (CB), leaving the hole in the VB [79]. Thus, the reduction reaction occurs in the CB, and the oxidation reaction occurs in the VB. Aryl halides were reduced in the CB to generate aryl radicals, which further perform hydrogenation or C-C coupling reactions [80]. Aryl halides can be reduced only when the CB potential of the semiconductor is more negative than that of the aryl halides [81]. Therefore, aromatic halide substrates with different reactivity can be reduced by semiconductors with different band positions. For example, ZnSe/CdS core-shell quantum dots could reduce 2-bromopyridine and 2-chloroquine under visible light conditions, and the yields were 76% and 49%, respectively [80]. When TiO_2_ and conjugated polymer poly(*p*-benzene) (PPP) were successfully used as multiphase co-catalyst (PPP/TiO_2_), electron-adsorbing group substituted aryl bromide/chlorine could be reduced for hydrodehalogenation under visible light conditions with triethylamine as an electron donor [82]. A new covalent triazine frameworks (CTF) containing phenanthroline fragments (Phen-CTF), as the visible light-mediated photocatalyst, showed an outstanding activity for the dehalogenation processes of aryl bromides and aryl chlorides with electron-withdrawing group to form C-C, C-P and C-B bonds [83]. Boron carbonitride (BCN) ceramics could reduce (het)aryl halides, including various substituted aromatic chlorides and aromatic bromides, to construct new C-H, C-C, and C-S bonds at ambient reaction conditions with visible light irradiation [84]. 

In addition, Semiconductors as photocatalysts loading on the noble metal nanoparticles with plasmonic resonance can significantly enhance visible absorption, reduce electron hole recombination, and promote the reduction of aryl halide substrates in CB [85,86,87]. For instance, 3%Ag/TiO_2_ could efficiently catalyze Suzuki coupling of various substituted bromobenzene and phenylboric acid to prepare biphenyl products under visible light condition [85]. Nano pd/ZnO could efficiently catalyze the reduction of aromatic iodine/bromine and electron-withdrawing aromatic chloride for cross-coupling, including Suzuki–Miyaura, Hiyama, and Buch–Wald–Hartwig reactions under visible light conditions [86]. Platinum nanoparticles on titania (PtNP@TiO_2_) could promote reductive dehalogenations of aryl iodides in the presence of diiso-propylethylamine ((*i*-Pr)_2_NEt) in moderate to excellent yields [87]. The mechanism study indicated that TiO_2_ decorated with 0.2% PtNP shows enhanced efficiency comparing with TiO_2_ alone, attributed to the platinum nanoparticles ability to both enhance the light absorption and inhibit recombination of electron–hole pairs.

## 5. Conclusions and Outlook

In the past decade, the reduction of aryl halides driven by visible light to aryl radicals develops rapidly. This is mainly due to the rapid development of visible-light-driven redox reactions. The old method relies on various stoichiometric reagents, such as tri-*n*-butyltin hydride (*n*-Bu_3_SnH) [88,89], while the visible-light-driven photocatalysis produces much less unnecessary and toxic products. In general, aryl radicals are versatile intermediates that can be obtained from inexpensive aryl halogen precursors under mild reaction conditions by visible-light-driven metal complexes, organic compounds, semiconductor catalysis, and alkali-assisted reduction in a series of systems. The application of aryl radicals in modern chemistry can be promoted due to the mildness of visible light catalytic system. However, there are also some problems in this field, such as the expensive price of catalyst, serious pollution, and difficult recovery of visible light homogeneous catalysts, and the photocatalytic efficiency also needs to be improved. Although semiconductors as photosensitizers are cheap; stable; and recyclable, noble metals, such as platinum and palladium, are mostly needed to load for the reductive dehalogenation of inert aromatic halides, and there is a problem of poor selectivity [81,85,87,90,91,92,93]. Therefore, looking for new pollution-free, low-cost, recyclable, and highly active catalysts and improving photo initiation efficiency are challenges that need to be solved in the next step in this research field.

## Data Availability

Not applicable.
